# Hearty Suppers

**Published:** 1882-08

**Authors:** 


**HEARTY SUPPERS.**—A case was recently stated in this JOURNAL, in which a clergyman rode from breakfast until night, without eating any thing. Weary and hungry, he ate a very hearty meal and retired to bed. During the night he was taken ill, fell at once into a stupor, and in that condition died next day. In another case, a man came into the hospital with both shoulders disjointed, the result of a hearty late supper, as there explained. In another instance, under our own observation, a person in as apparent good health as at any time during the fifty years preceding, was attacked with lung-fever, in consequence of hearty eating in the latter part of the afternoon, and died in three days. Usually, late and hearty suppers cause diarrhea, cholera, cramp-colic, and similar forms of disease, but in many cases their ill-effects are manifested in ways little suspected; hence they often get off with less than their share of blame. It is useful, therefore, to give some of their more uncommon results, this may lead a few of the wiser sort to adopt from principle, as a wise precaution, the safe, advantageous and rational practice of eating nothing later than the 6 P. M. meal, beyond a piece of cold bread and butter, adding, perhaps, not a glass of cold water, but what is better, a single teacupful of any hot drink. Lung-fever, inflammation of the lungs, and pneumonia, mean precisely the same thing, "Lung-Fever" being plain old Anglo-Saxon. The patient ate very heartily indeed, late in the after-part of the day, and waked up in the night with a severe chill, which shook the body like an aspen, and lasted for more than an hour; then came vomiting of bile, and looseness of bowels, and a little cough; these all suddenly ceased, and the patient sank rapidly into the grave, at the age of seventy-five. The lungs of this person had been so weak at the early age of twenty-two, that it was freely prophesied, that life could not last another year. When any thing is eaten, extra blood and heat go to the stomach to carry on the work of digestion, and this process ceases the instant the temperature is below nature's standard. An extra meal requires extra digestive power and extra heat; the blood is called in from the outposts, and so is the heat, to assist the stomach in its unusual labor: that leaves the surface, the skin, the feet, the fingers, cold. Has the reader never felt chills run over the body in getting up from a hearty meal? A greater degree of that would be a "regular shake." Now the lungs are in direct sympathy with the skin, hence the chill of the skin is often transferred to the lungs. In the case in question, the effects of the chill fell on the lungs, they being the weaker part, the chill of the skin drove all the blood inwards and congested it, heaped it on the incapable, the weak, the helpless lungs; as proof, the patient spat blood, a mouthful or less. It was nature's effort to get rid of the terrible load; if she had been able to clear herself of half a tea-cupful or more, the patient would have been saved, but there was not constitutional strength enough to make the effort; the clockwork of life had run so long, that all its wheels had pretty nearly worn out together, so that there was not power sufficient to overcome the obstacle of a pin-head, as it were. The main lesson of the article is, that eating heartily, late in the day, is always hurtful, sometimes dangerous and even fatal. And further, that safety in the old and the feeble consists in habitually guarding against even slight exposures, slight irregularities, and slight changes in any of the habits and practices of life, eating and drinking and exercising in the greatest moderation, being systematic and uniform in all things.

There are a few bodily ailments which are aggravated, and in some cases rendered incurable by insufficient diet; but with the exception of Diphtheria and a few others, nine out of ten of all ordinary ailments are controlled, are arrested, are permanently cured by a wise diminution of the amount of food eaten. This is particularly the case when there is no decided ailment, but a general feeling of discomfort or of unwellness. In all actively inflammatory maladies, where there is acute pain any where, total abstinence from all substantial food, from every thing liquid or solid, except hot teas, is the sheet-anchor of safety, when not extended beyond thirty-six hours. No one should venture on a longer abstinence on any occasion without the advice of a physician.

All pain is caused by over-distended blood-vessels pressing against some neighboring nerve. Hence the quickest way of relieving any ordinary pain is to diminish the amount of blood in the vessels of the part by bleeding. But there is a safer, a better and a more enduring relief in cutting off the supply of blood; and as blood is made out of the food we eat, it must be apparent, that if on the feeling of pain or discomfort, we cease eating absolutely, that pain must begin to diminish within six hours, that being the time required for converting food into blood, and if no more food is eaten, no more blood can be made; while the amount in the system is diminished at the rate of two or more pounds in every twenty-four hours of invalidism, there must be relief. But let it be remembered that while this diminution goes on in a state of rest by means of the perspiration, sensible and insensible, as well as by all the involuntary motions of the system, and the friction of the blood along its vessels, it is an important fact that every crook of the finger, every wink of the eye, every thought of the mind, is at the expense of the consumption of a greater or less number of particles of the body; so that, in every succeeding moment, the body weighs less than it did the preceding moment; this diminution takes place in the amount of the circulating fluids directly. If then the slightest motion diminishes the amount of blood, and it is the excess of blood in a part that causes pain, the next best means of diminishing pain, after cutting off the supply of food, is exercise. Hence the more a man exercises *short of actual fatigue*, the better he will feel, the sooner and more effectually will he be relieved. Many a time a man has felt uncomfortable, sometimes very decidedly so, but upon taking a walk or ride, or engaging in some interesting work, he expresses himself as having been greatly relieved. Let then this thought impress itself on the mind, that in the common every day ailments of life we must look for the cause in an excess of blood and other fluids in the body, and that whatever diminishes that excess is curative. The methods of this diminution are worth remembering. 1. Abstinence from food. 2. Perspiration, whether induced by covering up in bed and drinking hot teas, or by muscular exercise. 3. Vomiting. 4. Bleeding. 5. Counter-irritation, as by frictions with the hand or a mustard-plaster. Of these we recommend only abstinence, perspiration, exercise, friction. But let it be remembered that when exercise evidently increases the discomfort or the pain, or when it induces a positive feeling of weariness, it is contra-indicated, and quiet of mind and repose of body should be sedulously cultivated. And under all circumstances let it be remembered that however beneficial exercise may be in any given case. the very moment it becomes a felt fatigue, that moment it becomes a positive injury, if persisted in.

